# Vitamin D deficiency promotes accumulation of bioactive lipids and increased endocannabinoid tone in zebrafish

**DOI:** 10.1016/j.jlr.2021.100142

**Published:** 2021-10-18

**Authors:** Megan M. Knuth, Whitney L. Stutts, Morgan M. Ritter, Kenneth P. Garrard, Seth W. Kullman

**Affiliations:** 1 Lineberger Comprehensive Cancer Center, University of North Carolina School of Medicine at Chapel Hill, Chapel Hill, NC, USA; 2 Department of Genetics, University of North Carolina School of Medicine at Chapel Hill, Chapel Hill, NC, USA; 3 Toxicology Program, Department of Biological Sciences, North Carolina State University, Raleigh, NC, USA; 4 Molecular Education, Technology and Research Innovation Center (METRIC), North Carolina State University, Raleigh, NC, USA; 5 FTMS Laboratory for Human Health Research and Department of Chemistry, North Carolina State University, Raleigh, NC, USA; 6 Precision Engineering Consortium, Department of Mechanical and Aerospace Engineering, North Carolina State University, Raleigh, NC, USA; 7 Center for Human Health and the Environment, North Carolina State University, Raleigh, NC, USA

**Keywords:** MS, metabolomics, lipidomics, lipids, nutrition, endocannabinoid biosynthesis, MSI, IR-MALDESI, anandamide, 2-AG, 2-arachidonoylglycerol, *abh4*, alpha/beta hydrolase 4, AEA, anandamide, AT, adipose tissue, cDNA, complementary DNA, *cnr1*/CB1, cannabinoid receptor 1, *cnr2*/CB2, cannabinoid receptor 2, *dagla/b*, diacylglycerol lipase a/b, EC, endocannabinoid, *faah1*, fatty acid amide hydrolase 1, *faah2a*, fatty acid amid hydrolase 2, FDR, false discovery rate, *gde1*, glycerophosphodiester phosphodiesterase 1, GH, growth hormone, IR, insulin resistance, IR-MALDESI-MSI, infrared-matrix-assisted laser desorption electrospray ionization MS imaging, *mgll*, monoglyceride lipase, mpf, months post fertilization, *napepld*, *N*-acyl phosphatidylethanolamine phospholipase D, PC, phosphatidylcholine, PE, phosphatidylethanolamine, PPARγ, peroxisome proliferator-activated receptor gamma, *PPARGC1A*, proliferator activated receptor gamma coactivator 1α, qPCR, quantitative real-time PCR, ROI, region of interest, *trpv1*, transient receptor potential cation channel subfamily V member 1, TRPV1, transient receptor potential vanilloid 1, VDD, vitamin D deficiency, VDS, vitamin D-sufficient

## Abstract

Vitamin D is well known for its traditional role in bone mineral homeostasis; however, recent evidence suggests that vitamin D also plays a significant role in metabolic control. This study served to investigate putative linkages between vitamin D deficiency (VDD) and metabolic disruption of bioactive lipids by MS imaging. Our approach employed infrared-matrix-assisted laser desorption electrospray ionization MS imaging for lipid metabolite profiling in 6-month-old zebrafish fed either a VDD or a vitamin D-sufficient (VDS) diet. Using a lipidomics pipeline, we found that VDD zebrafish had a greater abundance of bioactive lipids (*N*-acyls, endocannabinoids [ECs], diacylglycerols/triacylglycerols, bile acids/bile alcohols, and vitamin D derivatives) suggestive of increased EC tone compared with VDS zebrafish. Tandem MS was performed on several differentially expressed metabolites with sufficient ion abundances to aid in structural elucidation and provide additional support for MS annotations. To confirm activation of the EC pathways, we subsequently examined expression of genes involved in EC biosynthesis, metabolism, and receptor signaling in adipose tissue and liver from VDD and VDS zebrafish. Gene expression changes were congruent with increased EC tone, with VDD zebrafish demonstrating increased synthesis and metabolism of anandamide compared with VDS zebrafish. Taken together, our data suggest that VDD may promote accumulation of bioactive lipids and increased EC tone in zebrafish.

Vitamin D (calciferol) is a prohormone traditionally associated with bone mineral homeostasis; however, recent research has implicated a role for vitamin D in additional physiological and cellular processes, including metabolic control, cell differentiation, oxidative stress, xenobiotic metabolism, neurodevelopment, and immune function (1, 2, 3, 4, 5, 6, 7, 8, 9). Leading to these discoveries are studies demonstrating that vitamin D deficiency (VDD) can result in dysregulation of essential cellular processes associated with autoimmune disease, poor bone health, and cardiometabolic diseases, such as obesity, insulin resistance (IR), and type 1 and type 2 diabetes (10, 11, 12, 13, 14, 15, 16).

In relation to human cardiometabolic functions, vitamin D status has been demonstrated to be inversely related to triglyceride and cholesterol levels, fasting blood glucose, and insulin (17, 18). Vitamin D status has also been demonstrated to impact body composition, with VDD individuals demonstrating greater waist circumferences and body fat percentages as seen in obese populations (17, 18). Children born to VDD mothers have metabolic profiles high in fatty acids (linoleate, linolenate, myristate, oleate, palmitate, and palmitoleate) and amines/derivatives (alanine, glutamate, hypoxanthine, lactate, phenylalanine, proline, and urea) known to be biomarkers of inflammation and IR (19, 20, 21). Thus, accumulating evidence suggests a link between vitamin D status and metabolic health, where VDD populations present elevated levels of fatty acid characteristic of cardiometabolic disease.

Fatty acids and fatty amines fall into the category of bioactive lipids. Bioactive lipids are endogenous signaling lipids known to play critical roles in cellular function by maintaining membrane integrity and have been implicated in cardiovascular disease, immune response, endocannabinoid (EC) tone, and inflammation (22, 23). Included in this category are bile acids, eicosanoids, docosanoids, sphingolipids, and ECs, to name a few. While not well investigated, preliminary studies suggest a potential linkage between VDD and metabolism of bioactive lipids including EC system modulation (24, 25). A recent study by Guida *et al.* (26) demonstrated elevated levels of the endogenous cannabinoid receptor (CB1) agonist anandamide (AEA) in the spinal cord of adult VDD mice compared with vitamin D normal controls. Stimulation of the EC system by cannabis has also been associated with reduced concentrations of serum 25(OH)D and poor bone mineral density in human populations, phenotypes characteristic of VDD (27). These preliminary data suggest that EC stimulation may be inversely correlated with vitamin D levels.

Activation of the EC system has been directly linked to fat storage and weight gain (28). Mice treated with AEA during lactation demonstrated increased body fat content and hypertrophy of adipose tissue (AT) directly associated with elevated levels of CB1 (29). Zebrafish cannabinoid receptor mutants (*cnr1*^*−/−*^/*cnr2*^*−/−*^) support such a relationship where deactivation of the EC system was directly associated with reduced *srefb1* (sterol regulatory element-binding transcription factor 1) gene expression (30). This study further linked disrupted EC signaling to impaired liver development, hepatic differentiation, and proliferation (30). In contrast, zebrafish treated with AEA demonstrated enhancement of *srebf1* expression known to promote hepatic lipid accumulation, lipotoxicity, and enhancement of insulin-like growth factors (*igf1*/*igf2*) known to promote adipocyte proliferation, differentiation, and deposition, suggesting that activation of the EC system may promote lipogenic processes (31, 32, 33, 34).

Previously, our laboratory established a linkage between VDD and metabolic dyshomeostasis, where low levels of vitamin D resulted in stunted growth and central adiposity in zebrafish by 6 months post fertilization (mpf) (35). The purpose of the study presented herein was to further investigate this linkage using a spatial lipidomics approach and identify putative differences in untargeted lipid metabolites between VDD and VDS zebrafish at 6 mpf. Infrared-matrix-assisted laser desorption electrospray ionization MS imaging (IR-MALDESI-MSI) was used to determine altered lipid metabolite profiles, spatial distributions, and abundance between VDD and VDS in whole sectioned zebrafish (36, 37).

In this study, we focused on metabolites that were more abundant in VDD zebrafish than VDS zebrafish. Our assessments covered a region of interest (ROI) that included AT, liver, intestines, spleen, and kidney. Select ions across both positive and negative ionization modes were identified as having established roles in EC signaling. Confirmatory gene expression data indicated that EC biosynthesis and metabolism are highly dysregulated in our VDD zebrafish. Collectively, results from this study suggest a putative linkage between VDD and EC signaling as a putative mechanism of metabolic dyshomeostasis.

## Materials and methods

### Zebrafish husbandry and maintenance

All zebrafish were maintained as previously described, according to the protocols approved by the North Carolina State University Institutional Animal Care and Use Committee (35). Adult zebrafish were maintained at appropriate densities in 9 L tanks as part of a recirculating aquatics system under a 14:10 h light:dark cycle. Water temperature was maintained at 28.5 (±0.5)°C with a pH between 6.8 and 7.5. To ensure that VDD zebrafish did not exchange water with VDS fish, they were kept in their own quarantine system set to the same parameters as the VDS fish.

### Cohort generation

Dietary cohorts were generated as previously described with a few modifications (35). To generate F0 cohorts, ABxAB zebrafish were raised on a standard larval laboratory diet (11.5 IU/g VD) until 2 months of age. At the 2-month time point, juvenile zebrafish were assessed for standard length to determine the average standard length for the population, and any fish ±2 standard deviations away from the mean were removed from the cohort. The remaining fish were equally divided and placed into six, 9 L, mixed-gender tanks. The tanks were grouped in two sets of three. Upon transfer into their new housing, the 2-month-old zebrafish began their new diet: VDD diet (0 IU/g) or VDS diet (400,000 IU/g) (supplemental Table S1). The zebrafish were kept on their designated engineered diet throughout the rest of their life span. Only male zebrafish were used in the following studies to eliminate the impact of sex differences on VDD and adiposity.

### Sample preparation and cryosectioning for IR-MALDESI-MSI

All samples were prepared as previously described (37). Adult male zebrafish underwent a 24-h starvation prior to the morning of the experiment and were euthanized in cold water. A 5% carboxymethylcellulose + 10% gelatin matrix was prepared and maintained in an ∼80°C water bath to prevent solidification. Fish were embedded once the gel mix had been in the water bath for approximately 15 min. Fish were embedded as previously described and sectioned using a cryostat (Leica CM 1950; Leica Biosystems, Buffalo Grove, IL) cooled to −20°C (37). The embedded fish were flash frozen in a dry ice/ethanol bath for approximately 15 min until the entire matrix was completely frozen, and the embedded fish could be mounted on the specimen disk. Parasagittal sections, 16 μm thick, were collected from each zebrafish at the ROI and thaw mounted onto glass microscope slides (Thermo Fisher Scientific, Bremen, Germany) for IR-MALDESI-MSI.

### IR-MALDESI-MSI

**Fig. 1:**
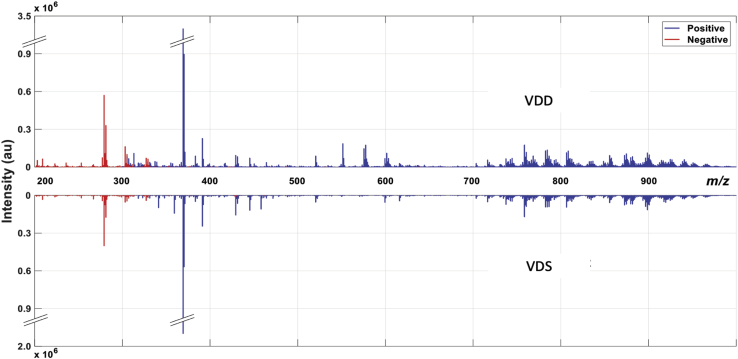
Comparison of ROI lipid profiles from a VDD and VDS zebrafish. Mirror plots from full-scan MS^1^ spectra illustrate the chemical diversity and changes in lipid composition observed in VDD fish.

IR-MALDESI experiments utilized a 2,940 nm mid-IR laser (IR-Opolette 2371; Opotek, Carlsbad, CA) with a pulse frequency of 20 Hz as described by Stutts *et al.* (37). All data were acquired using a laser shot spacing of 100 μm, approximately equal to the laser ablation spot diameter, providing a spatial resolution of ∼100 μm. Each spectrum was acquired using one laser pulse per voxel and a 25 ms maximum ion injection time. The electrospray solvent composition was 50:50 (v/v) methanol:water with 0.2% formic acid, and a flow rate of 1–1.5 μl/min was optimized for spray stability on the day of analysis. High-resolution accurate mass measurements were acquired in both positive and negative ionization modes at a resolving power of 140,000 (full width at half maximum, *m/z* 200) using a Q Exactive Plus mass spectrometer (Thermo Fisher Scientific, Bremen, Germany). The scan range was set to *m/z* 300–1,200 and *m/z* 200–800 for positive and negative ionization modes (Fig. 1), respectively, and the S-lens RF level was set at 50%. For positive mode data acquisition, the electrospray voltage was set at 3.8 kV and the capillary temperature at 320°C; for negative mode, these parameters were 3.2 kV and 275°C.

### Histology

Serial sections from the IR-MALDESI-MSI cryosectioning protocol were fixed in 10% neutral buffered formalin and processed for H&E staining as previously described (37). Images were acquired with a 5× magnification on an LMD7000 (Leica, Buffalo Grove, IL).

### Data analysis

#### Pipeline A

**Fig. 2:**
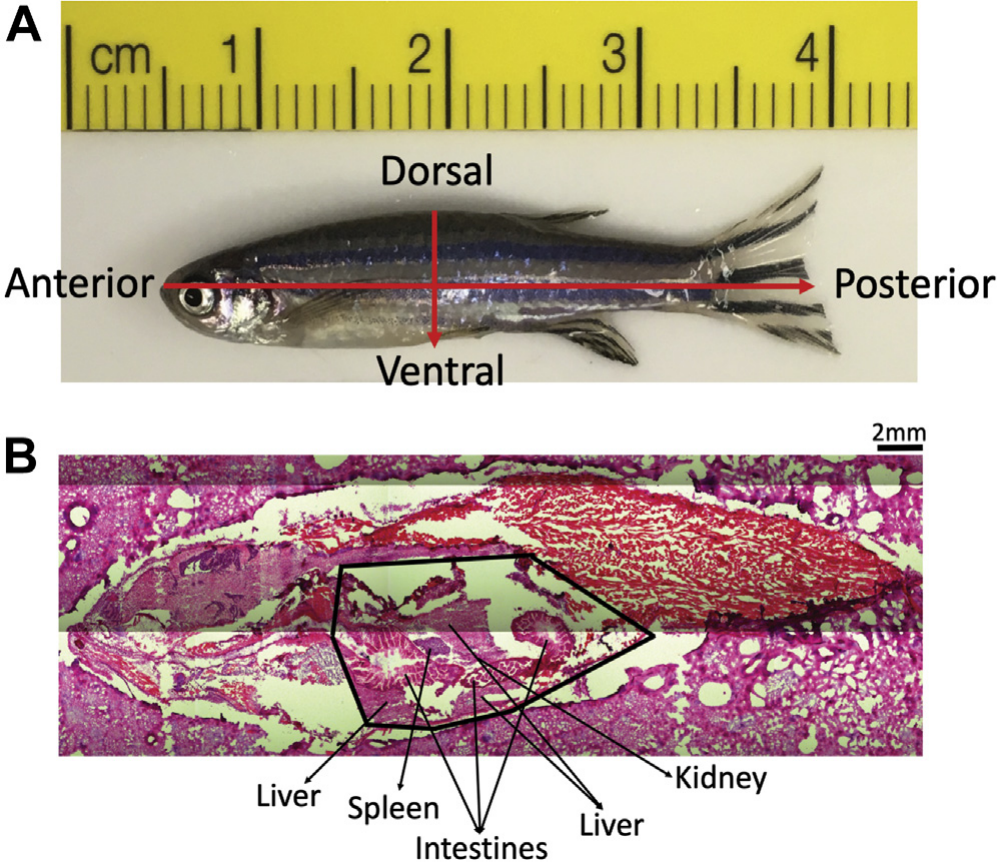
Zebrafish orientation. A: All fish shown in IR-MALDESI-MSI images are presented in anterior to posterior and dorsal to ventral orientation. B: Representative histology image of a VDD fish with the visceral cavity (liver, intestines, spleen, and kidney) labeled.

**Supplemental Figure 1:**
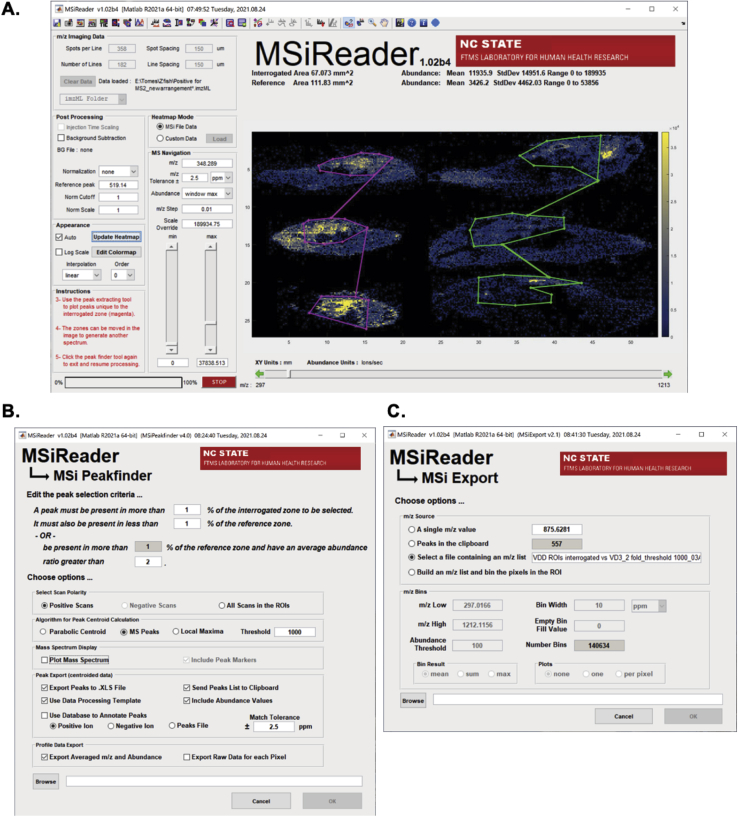


Separately for both positive and negative ionization mode data, imzML folders containing all six fish from both treatment groups were loaded into MSiReader, version 1.02b4 using a minimum abundance threshold of 1,000 (38, 39). Images were loaded as two columns and three rows. For visualizing whole-body images, the *m/z* value set in the “MS Navigation” box for positive mode was 348.2890 ± 2.5 ppm and for negative mode was 279.2329 ± 2.5 ppm. Once the images were loaded into MSiReader, several export tools were used in order to process the data for statistical analysis. To start, the “Enable polygon tool to create interrogated and reference zones” was selected. VDD fish were selected as the interrogation group, whereas VDS fish were selected as the reference group. Rather than investigating differences across the entire fish and risking the impact of off-target metabolites, a defined ROI was selected within each of the six fish. Here, we established a broad ROI encapsulating the central visceral cavity (AT, liver, intestines, spleen, and kidney) (Fig. 2B, and supplemental Fig. S1A). To ensure all tissues of interest were included in each ROI, histological slides were referenced during the outlining of the ROIs in MSiReader. Next, the “Extract peaks that are unique to the interrogated zone” tool was selected, and all peaks 2-fold or greater in the VDD fish ROIs than the VDS fish ROIs were extracted (supplemental Fig. S1B). Once the export was complete, the “Enable polygon tool for ROI selection” was used to select all pixels, and abundance data were exported using the “Export abundance data for selected pixels” tool. Within the MSi Export window, the *m/z* file containing peaks unique to the interrogated zone was loaded (supplemental Fig. S1C). The resulting export file contained pixel-by-pixel abundance data for all peaks 2-fold or greater in the VDD fish ROIs. Next, the pixel-by-pixel abundance data for each peak was averaged for each fish, and a peak intensity table was created in a new Excel file containing seven columns: one column for peaks (*m/z*), three columns of VDD fish abundance data, and three columns of VDS fish abundance data. To determine ions of significant difference between treatment groups, a standard equal variance *t*-test was conducted in Excel followed by a fold-change analysis. All ions with a *P* value ≤0.05 (∗∗) or a *P* value ≤0.1 (∗) were considered significant. Lipid class and putative ion identities were determined using the METASPACE annotation platform and the METLIN Mass Spectral Database simple and advanced search functions (40, 41). Twelve imzML-formatted data files were uploaded into METASPACE, and false discovery rate (FDR)-controlled annotations were obtained by searching the Human Metabolome Database (version 4) and LIPID MAPS database (LipidMaps 2017-12-12) for [M + H]^+^, [M + Na]^+^, [M + NH_4_]^+^, and [M + K]^+^ (positive mode data) and [M − H]^−^ and [M + Cl]^−^ (negative mode data). METLIN was searched using these same adducts and a mass accuracy tolerance of 3 ppm. A series of literature reviews followed in order to classify ions as bile acids/alcohols, *N*-acyls, vitamin D derivatives, diacylglycerides, triacylglycerides, or lipids involved in EC signaling. The resulting ions are represented in Tables 1 and 2.

**Table 1:** Negative mode ions detected by IR-MALDESI-MSI as significantly more abundant in VDD zebrafish following data analysis pipeline A^a^

Negative Mode Ions					
Putative Ion Identity^a^	Chemical Formula	Ion Type	Measured *m/z* ± 2.5 ppm	Fold Change	*P*
Lipids involved in EC signaling					
Arachidonic acid^b^^,^^c^	C_20_H_32_O_2_	[M − H]^−^	303.2333	2.14	d
Eicosatrienoic acid^c^	C_20_H_34_O_2_	[M − H]^−^	305.2489	4.56	e
Adrenic acid^c^	C_22_H_36_O_2_	[M − H]^−^	331.2644	2.63	e
Docosatrienoic acid^c^	C_22_H_38_O_2_	[M − H]^−^	333.2801	2.80	d
Others					
5α-Androstane^c^	C_19_H_32_	[M − H]^−^	259.2436	3.14	d
Nonadecanoic acid^c^	C_19_H_36_O_2_	[M − H]^−^	295.2639	2.87	e
Sorbitan palmitate^c^	C_22_H_42_O_6_	[M − H]^−^	401.291	3.10	e

Putative ion identities and chemical formulas were determined for negative mode ions following data analysis pipeline A. These ions were significantly (*P* ≤ 0.1; *P* ≤ 0.05) more abundant (2-fold or greater) in VDD than VDS zebrafish at 6 mpf.

a Isomers are not resolved and may contribute to the observed abundances.

b MS^2^ match for chemical formula.

c The ion is considered a bioactive lipid.

d *P* ≤ 0.1.

e *P* ≤ 0.05.

**Table 2:** Positive mode ions detected by IR-MALDESI-MSI as significantly more abundant in VDD zebrafish following data analysis pipeline A^a^

Positive Mode Ions					
Putative Ion Identity^a^	Chemical Formula	Ion Type	Measured *m/z* ± 2.5 ppm	Fold Change	*P*
Lipids involved in EC signaling					
*N*-oleoyl ethanolamine^b^	C_20_H_39_NO_2_	[M + H]^+^	326.3053	2.30	c
*N*-ethyl arachidonoyl amine^b^	C_22_H_37_NO	[M + H]^+^	332.2946	3.76	c
Anandamide (20:4, n-6)^b^	C_22_H_37_NO_2_	[M + H]^+^	348.2896	3.62	c
Tricosahexaenoic acid^b^^,^^d^	C_23_H_34_O_2_	[M + NH_4_]^+^	360.2898	6.05	c
Anandamide (22:6, n-3)^b^	C_24_H_37_NO_2_	[M + H]^+^	372.2903	2.87	e
Arachidonoylmorpholine^b^	C_24_H_39_NO_2_	[M + H]^+^	374.3061	5.05	c
*N*-(1,1-dimethyl-2-hydroxy-ethyl) arachidonoyl amine^b^	C_24_H_41_NO_2_	[M + H]^+^	376.3213	3.77	c
*N*-arachidonoyl GABA^b^	C_24_H_39_NO_3_	[M + H]^+^	390.3011	6.17	c
*N*-(5-hydroxy-pentyl) arachidonoyl amine^b^	C_25_H_43_NO_2_	[M + H]^+^	390.3369	6.54	c
Phosphatidylcholine (42:8)^b^	C_50_H_84_NO_8_P	[M + NH_4_]^+^	875.6281	5.78	c
Phosphatidylcholine (44:11)^b^	C_52_H_82_NO_8_P	[M + NH_4_]^+^	897.6097	4.64	e
*N*-Acyls					
*N*-methyl arachidonoyl amine^b^	C_21_H_35_NO	[M + H]^+^	318.2796	5.68	c
*N*-oleoyl praline^b^	C_23_H_41_NO_3_	[M + H]^+^	380.3157	3.11	c
*N*-Palmitoyl phenylalanine^b^	C_25_H_41_NO_3_	[M + H]^+^	404.3166	9.80	c
*N*-oleoyl phenylalanine^b^	C_27_H_43_NO_3_	[M + H]^+^	430.3326	8.07	e
Diacylglycerols/triacylglycerols					
Diacylglycerol (32:0)^b^	C_35_H_68_O_5_	[M + NH_4_]^+^	586.5409	4.59	c
Diacylglycerol (34:1)^b^	C_37_H_70_O_5_	[M + NH_4_]^+^	612.5563	2.55	c
Diacylglycerol (37:2)^b^	C_40_H_74_O_5_	[M + NH_4_]^+^	652.5884	2.85	c
Diacylglycerol (37:1)^b^	C_40_H_76_O_5_	[M + NH_4_]^+^	654.6029	7.90	c
Triacylglycerol (48:2)^b^	C_51_H_94_O_6_	[M + NH_4_]^+^	820.738	8.30	c
Triacylglycerol (48:1)^b^^,^^d^	C_51_H_96_O_6_	[M + NH_4_]^+^	822.7554	18.29	c
Triacylglycerol (48:0)^b^	C_51_H_98_O_6_	[M + NH_4_]^+^	824.7707	6.32	e
Triacylglycerol (50:1)^b^	C_53_H_100_O_6_	[M + H]^+^	833.7616	4.90	e
		[M + NH_4_]^+^	850.7884	2.86	e
Triacylglycerol (51:1)^b^	C_54_H_102_O_6_	[M + NH_4_]^+^	864.8038	7.82	e
Triacylglycerol (53:3)^b^	C_56_H_102_O_6_	[M + NH_4_]^+^	888.8029	2.50	c
Triacylglycerol (53:2)^b^	C_56_H_104_O_6_	[M + NH_4_]^+^	890.8164	3.44	e
Triacylglycerol (53:1)^b^	C_56_H_106_O_6_	[M + NH4]^+^	892.8342	8.35	e
Triacylglycerol (55:3)^b^	C_58_H_106_O_6_	[M + NH_4_]^+^	916.8338	2.64	c
Triacylglycerol (55:2)^b^	C_58_H_108_O_6_	[M + NH_4_]^+^	918.8481	3.68	e
Triacylglycerol (61:8)^b^	C_64_H_108_O_6_	[M + NH_4_]^+^	990.8505	2.81	e
Sterols					
5α-Cholan-24-oic acid^b^	C_24_H_40_O_2_	[M + NH_4_]^+^	378.3363	2.86	c
Vitamin D3 derivative	C_27_H_42_O_2_	[M + H]^+^	399.3254	4.33	e
Vitamin D3 derivative	C_26_H_42_O_3_	[M + NH_4_]^+^	420.3466	6.88	e
Vitamin D3 derivative	C_26_H_44_O_3_	[M + NH_4_]^+^	422.3619	6.93	e
Vitamin D2 derivative	C_28_H_44_O_2_	[M + NH_4_]^+^	430.3682	8.75	e
Vitamin D3 or cholesterol derivative^d^	C_27_H_46_O_4_	[M + H]^+^	435.3473	4.42	e
		[M + NH_4_]^+^	452.3732	6.01	e
5b-Cholestane-3a,7a,12a,24,25-pentol^b^^,^^d^	C_27_H_48_O_5_	[M + NH_4_]^+^	470.3843	9.02	c
Vitamin D3 derivative	C_29_H_50_O_4_	[M + NH_4_]^+^	480.4049	3.74	e
1a,25-dihydroxy-2b-(3-hydroxypropoxy)-19-norvitamin D3^d^	C_29_H_50_O_5_	[M + NH_4_]^+^	496.3988	7.58	c
Vitamin D3 derivative	C_31_H_52_O_4_	[M + NH_4_]^+^	506.4215	3.10	c
Vitamin D3 derivative	C_31_H_52_O_5_	[M + NH_4_]^+^	522.4157	7.46	c
Vitamin D3 derivative	C_31_H_54_O_5_	[M + NH_4_]^+^	524.4315	8.02	c
5β-Cyprinolsulfate^b^	C_27_H_48_O_8_S	[M + NH_4_]^+^	550.3424	5.68	e
Cholesteryl beta-d-glucoside^b^^,^^d^	C_33_H_56_O_6_	[M + NH_4_]^+^	566.4412	3.25	c
Cholesteryl palmitate^b^	C_43_H_76_O_2_	[M + NH_4_]^+^	642.6189	4.18	e
Others					
Ceramide^b^	C_35_H_69_NO_3_	[M + H]^+^	552.5357	12.94	e
Sphingomyelin^b^	C_38_H_77_N_2_O_6_P	[M + H]^+^	689.5586	2.93	c
C20 sulfatide^b^	C_44_H_85_NO_11_S	[M + H]^+^	836.5918	2.10	c
C22 sulfatide^b^	C_46_H_89_NO_11_S	[M + H]^+^	864.6225	3.60	e

Putative ion identities and chemical formulas were determined for positive mode ions following data analysis pipeline A. These ions were significantly (*P* ≤ 0.1; *P* ≤ 0.05) more abundant (2-fold or greater) in VDD than VDS zebrafish at 6 mpf.

a Isomers are not resolved and may contribute to the observed abundances.

b The ion is considered a bioactive lipid.

c *P* ≤ 0.05.

d MS^2^ match for chemical formula. The ion underwent MS^2^ analysis.

e *P* ≤ 0.1.

#### Pipeline B

After identifying a broad sweep of *N-*acyls (bioactive lipids) involved in EC signaling, we explored the LIPID MAPS Structural Database for fatty amides (42). We searched for the protonated ions of these known fatty amides in our dataset and compiled the list of biologically relevant ions found (Table 3). These ions were more abundant in our VDD zebrafish than the VDS, and although they did not meet our criteria for fold change (2-fold) and significance (*P* < 0.10), they strengthened our overall findings.

**Table 3:** Putative fatty amides detected by IR-MALDESI-MSI and elevated in VDD zebrafish viscera following data analysis pipeline B^a^

Positive Mode Ions					
Putative Ion Identity^a^	Chemical Formula	Ion Type	Measured *m/z* ± 2.5 ppm	Fold Change	*P*
ECs (fatty acids)					
Anandamide (18:3, n-6)^b^	C_20_H_35_NO_2_	[M + H]^+^	322.2741	1.90	c
Anandamide (18:2, n-6)^b^	C_20_H_37_NO_2_	[M + H]^+^	324.2902	1.73	c
Anandamide (20:5, n-3)^b^	C_22_H_35_NO_2_	[M + H]^+^	346.2751	1.61	0.1351
Anandamide (20:3, n-3)^b^	C_22_H_39_NO_2_	[M + H]^+^	350.3051	1.79	c
*N*-palmitoyl dopamine^b^	C_24_H_41_NO_3_	[M + H]^+^	392.3168	5.39	0.1814
Tetracosahexaenoylethanolamide^b^	C_26_H_41_NO_2_	[M + H]^+^	400.3207	2.87	0.2405
*N*-(16,16-dimethy-5Z,8Z,11Z,14Z-docosatetraenoyl)-ethanolamine^b^	C_26_H_45_NO_2_	[M + H]^+^	404.3533	8.60	0.1167
*N*-oleoyl dopamine^b^	C_26_H_43_NO_3_	[M + H]^+^	418.3324	7.43	0.1525
*N*-Acyls					
*N*-arachidonoyl glycine^b^	C_22_H_35_NO_3_	[M + H]^+^	362.2687	2.41	0.1806
*N*-amyl arachidohoyl amine^b^	C_25_H_43_NO	[M + H]^+^	374.3427	6.97	0.1716
*N*-arachidonoyl dihydroxypropylamine^b^	C_23_H_39_NO_3_	[M + H]^+^	378.3001	3.96	0.1209
*N*-propyl-16,16-dimethyl-5Z,8Z,11Z,14Z-docosatetraenoyl amine^b^	C_27_H_47_NO	[M + H]^+^	402.3737	2.38	0.1893

Additional putative ion identities and chemical formulas were determined for positive mode ions following data analysis pipeline B. These ions were more abundant in VDD than VDS zebrafish at 6 mpf.

a Isomers are not resolved and may contribute to the observed abundances.

b The ion is considered a bioactive lipid.

c *P* ≤ 0.1.

### IR-MALDESI-MS^2^

IR-MALDESI-MS^2^ analyses were performed on selected ions of interest to obtain additional confidence in putative identifications. Instrument parameters were the same as described previously with the following modifications for MS^2^ experiments. To maximize sensitivity, two laser pulses were used for desorption with a maximum ion injection time of 110 ms. The electrospray voltage was set at 3.5 and 3.8 kV for positive and negative modes, respectively, and the capillary temperature was set at 400°C. Precursor ions were isolated with a 0.9–1.5 Da window and fragmented using higher energy collisional dissociation. Normalized collision energies ranging from 10 to 30% were used to induce fragmentation. The scan range for each ion was adjusted to include the precursor ion and fragment ions as low as 50 Da or 100 Da for ions less than or greater than 800 Da, respectively. Mass spectra were analyzed using Xcalibur, version 4.2.28.14 (Thermo Scientific, Bremen, Germany) and fragment ion, centroided mass lists were obtained using MSiReader, version 1.02b2 (abundance threshold set to 10 and an *m/z* bin width of 10 ppm) and crossreferenced with MetFrag In silico fragmentation database (43). MS^2^ peak lists were searched against Kyoto Encyclopedia of Genes and Genomes, Human Metabolome Database, and LIPID MAPS using a relative mass deviation of 5 ppm and absolute mass deviation of 0.001 Da. Matching fragment ions were used to support chemical formula assignments and guide compound annotation in conjunction with METLIN, METASPACE, and literature review.

### Quantitative real-time PCR

Quantitative real-time PCR (qPCR) was used to measure targeted gene expression in male zebrafish liver and AT at 6 mpf as previously described (35). Complementary DNA (cDNA) was synthesized from total RNA using 10× random primers, 10× reverse transcription buffer, MultiScribe Reverse Transcriptase, and 10 mM deoxynucleotide triphosphates from a High Capacity cDNA Reverse Transcription Kit (Applied Biosystems, Foster City, CA) along with RNasin(R) RNase Inhibitor (Promega, Madison, WI). Primer sequences were designed using Primer3web, version 4.1.0. Primers were ordered from Integrated DNA Technologies, Inc (Coralville, IA). See supplemental Table S2 for a full list of primer sequences. Gene expression patterns were quantified using an Applied Biosystems 7300 real-time PCR machine. Biological replicates (n = 3–4/diet) were plated in triplicates and amplified in a 96-well clear Olympus PCR plate (Genesee, Morrisville, NC). Each well contained a 20 μl mixture of UltraPure water (Invitrogen, Marietta, OH), forward primer, reverse primer, cDNA, and iTaq Universal SYBR Green Supermix (Bio-Rad, Hercules, CA). Each reaction occurred under the following conditions: *1*) 50°C for 2 min, *2*) 95°C for 10 min, and *3*) 95°C for 15 s followed by 60°C for 1 min (repeated 40 times). This cycle was followed by a dissociation stage, which ensured primer specificity and confirmed the absence of primer dimerization: *4*) 95°C for 15 s, 60°C for 1 min, 95°C for 15 s, and 60°C for 1 min. Individual threshold cycle values (C_t_) were determined for each reaction by the ABI 7300 System SDS Software, and relative fold-change differences for each gene across each sample were calculated according to the ΔΔC_t_ method (44, 45). Gene expression was normalized to *efla* as the housekeeping gene.

## Results

### IR-MALDESI-MSI

To investigate the linkage between VDD and systemic metabolic disruption, IR-MALDESI-MSI was utilized for whole-body global lipid profiling (Fig. 1). Over 2,400 metabolites and lipids were annotated with an FDR <10% after filtering for “on-sample only” ions classified using a machine learning model in METASPACE (46). Candidate lipid metabolites from both negative and positive ionization modes were analyzed in MSiReader, version 1.02b4 (supplemental Fig. S1), METLIN, and METASPACE. For this study, we chose to focus only on ions found to be significantly (∗*P* ≤ 0.1; ∗∗*P* ≤ 0.05) more abundant in the VDD fish visceral ROI (AT, liver, intestines, spleen, and kidney) than the VDS fish (Tables 1 and 2). Using a high resolving power mass spectrometer, many isobaric compounds were resolved, allowing for the confident assignment of chemical formulae using accurately measured masses (<3 ppm mass accuracy) and relative ion abundances within an isotopic envelope (spectral accuracy). However, in some cases, the putative ion identities or compound names may be confounded by isomeric ions, which were not resolved. All isomers and potential isobars that are represented in the METASPACE databases searched appear in the publicly available results along with corresponding FDR and Metabolite-Signal Match score (https://metaspace2020.eu/project/Knuth_et_al_2021). Upregulated ions putatively identified in negative mode (Table 1) were classified as bioactive lipids, including arachidonic acid, eicosatrienoic acid, adrenic acid, and docosatrienoic acid, which function in EC biosynthesis/metabolism (Table 1). In positive mode (Table 2), we not only putatively identified additional bioactive lipids but also detected several forms of AEA, the well-characterized EC agonist (Tables 2 and 3). Of note, various ammonium adducts were observed, particularly for neutral lipids, although a source of ammonium was not added. Many of these ammonium adducts have been previously reported, including those of triglycerides, using the same or similar ionization methods (47, 48, 49, 50). Two possible theories for these adducts are given by Zhou *et al.* (47) including desorption of in situ ammonium from tissues or thermal decomposition of amino acids during laser ablation. The source of ammonium is unknown in this study, but the possibility of increased amines in these tissues following VDD is of interest. Of the ions putatively identified as being upregulated in VDD viscera, the majority were classified as bioactive lipids known to function in EC biosynthesis, metabolism, and signaling.

**Fig. 3:**
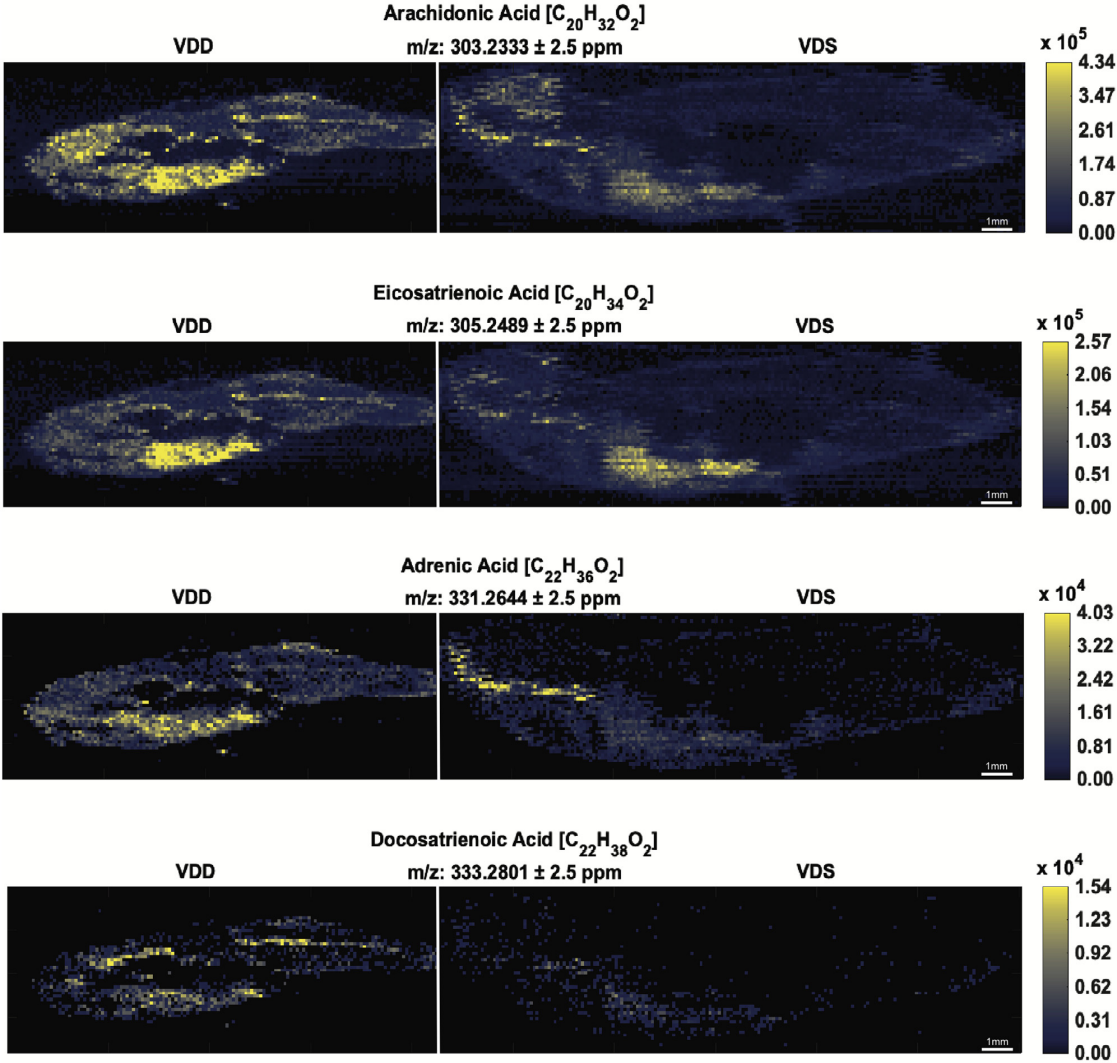
MSiReader images representing a select group of EC precursors detected in negative ionization mode. Fish are in anterior to posterior, dorsal to ventral orientation. Each image contains two male zebrafish at 6 mpf: a VDD fish (left) followed by a VDS fish (right). All ions were significantly (∗*P* ≤ 0.1; ∗∗*P* ≤ 0.05) more abundant (2-fold or greater) in VDD fish than VDS fish. ∗See also Table 1.

**Fig. 4:**
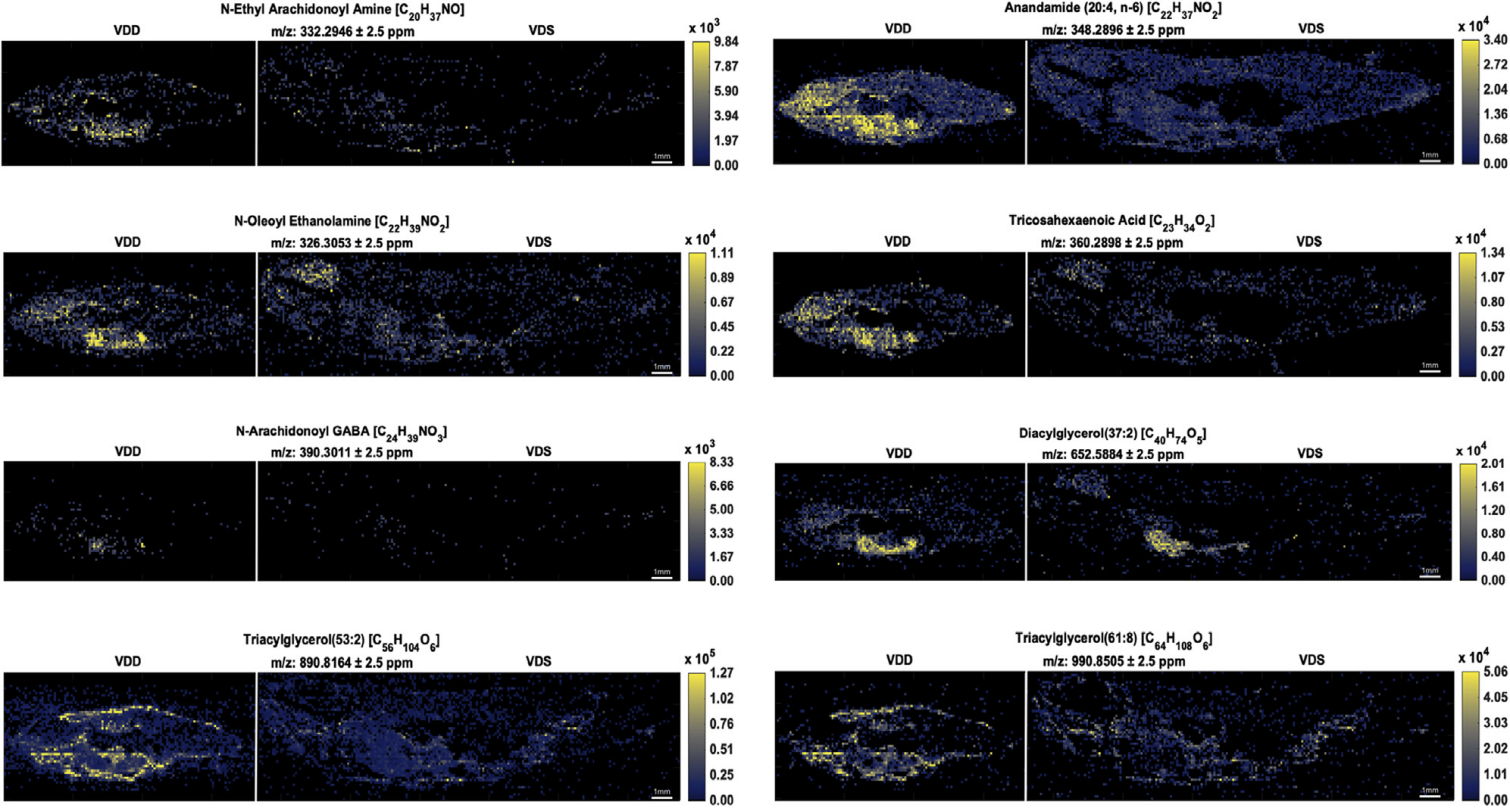
MSiReader images representing a select group of EC ions detected in positive ionization mode. Fish are in anterior to posterior and dorsal to ventral orientation. Each image contains two male zebrafish at 6 mpf: a VDD fish (left) followed by a VDS fish (right). All ions were significantly (∗*P* ≤ 0.1; ∗∗*P* ≤ 0.05) more abundant (2-fold or greater) in VDD fish than VDS fish. ∗See also Table 2.

**Fig. 5:**
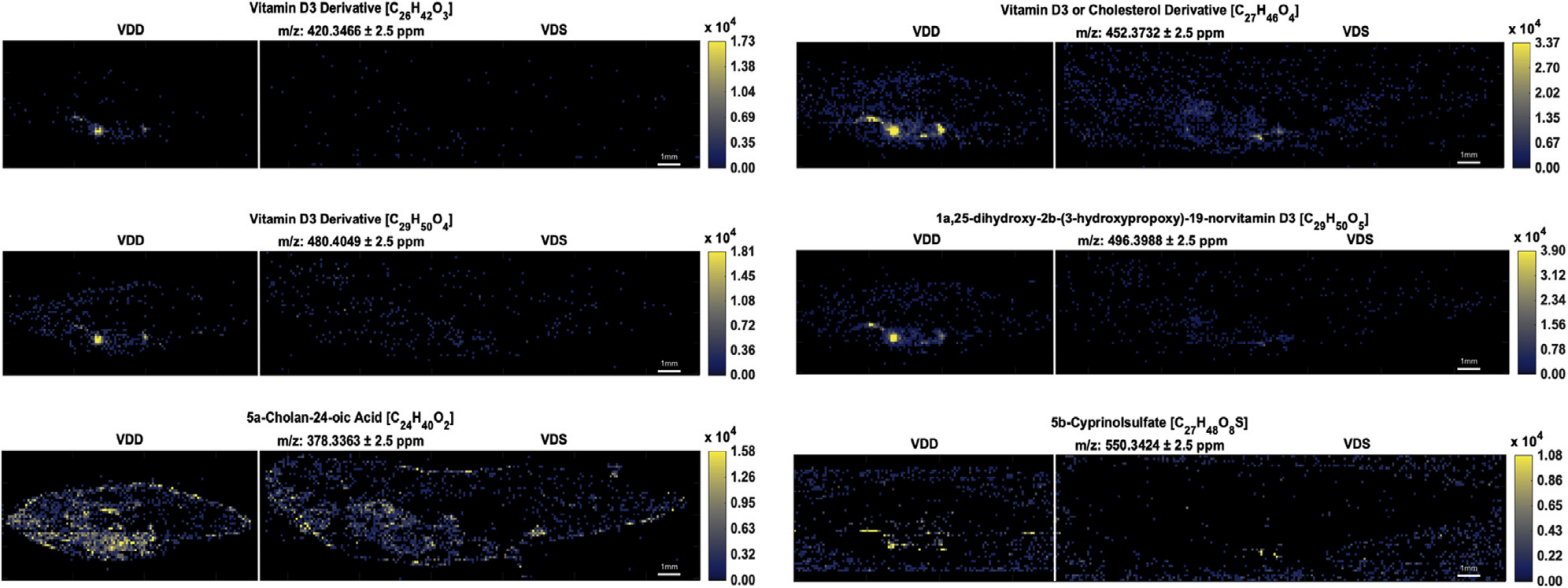
MSiReader images representing a select group of sterols in positive ionization mode. Fish are in anterior to posterior and dorsal to ventral orientation. Each image contains two male zebrafish at 6 mpf: a VDD fish (left) followed by a VDS fish (right). All ions were significantly (∗*P* ≤ 0.1; ∗∗*P* ≤ 0.05) more abundant (2-fold or greater) in VDD fish than VDS fish. ∗See also Table 2.

Analysis of lipid localization using MSiReader determined distinct spatial distribution and tissue localization of lipid classes. All fish shown in representative images are in dorsal-ventral and anterior-posterior orientation (Fig. 2). In negative mode, all ions visually represented (Fig. 3) are known fatty acyl precursors to ECs and distribute predominantly throughout the visceral cavity. In positive mode, all ions predominantly localized within the visceral cavity (Fig. 4), except for the triacylglycerols that demonstrated a more distinct distribution pattern throughout the subcutaneous AT and skin (Fig. 4). All representative vitamin D derivatives were nearly absent in the VDS fish and tightly localized within the visceral cavity of the VDD fish (Fig. 5), indicating a putative compensatory mechanism for vitamin D synthesis. Of the two bile acids/alcohols represented (Fig. 5), 5α-cholan-24-oic acid demonstrated localization patterns similar to the triacylglycerols observed in Fig. 4, and 5β-cyprinolsulfate showed localization similar to the vitamin D derivatives observed in Fig. 5.

**Fig. 6:**
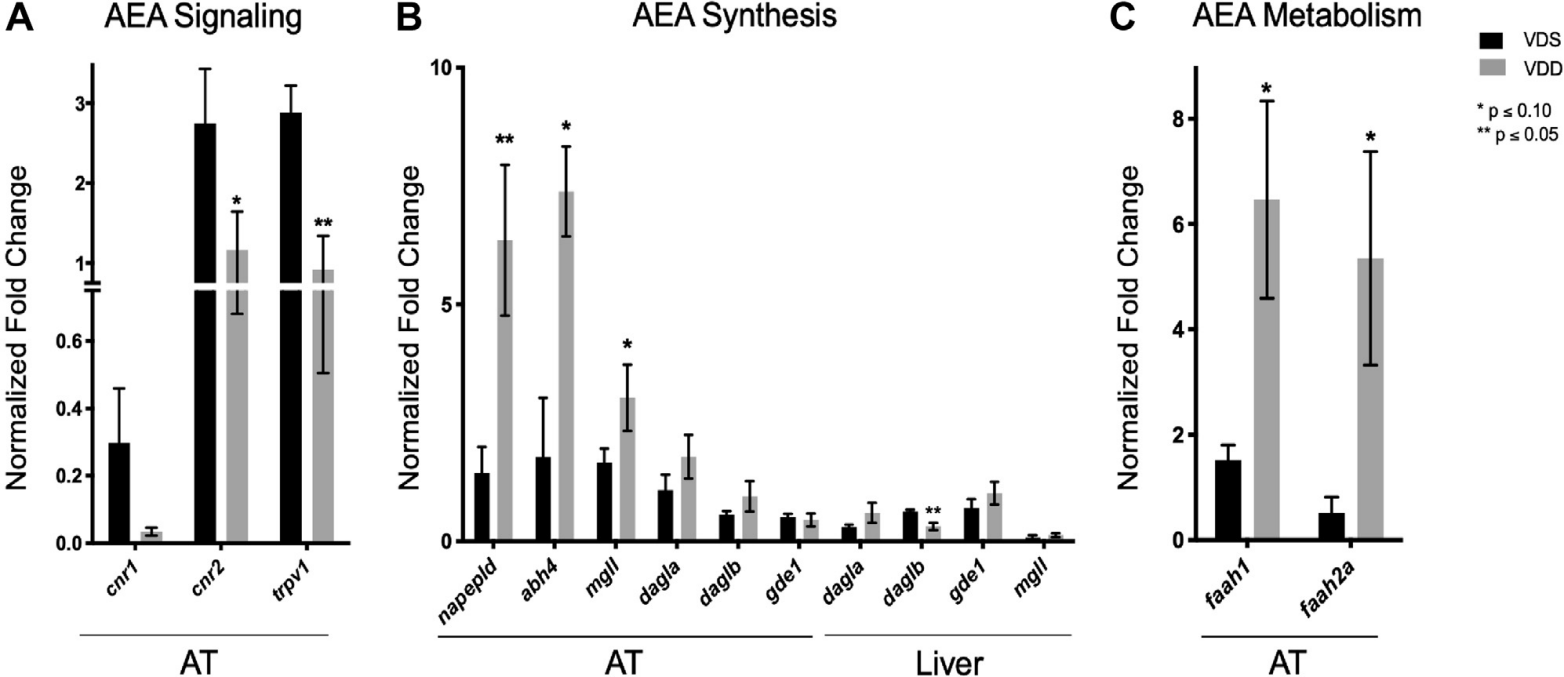
Evaluating AEA signaling, synthesis, and metabolism in VDD AT and Liver. A: VDD fish had significantly attenuated *trpv1* and *cnr2* expression and attenuated expression of *cnr1* in AT compared with VDS fish. B: VDD fish had significantly increased *napepld*, *abh4*, and *mgll* expression in AT compared with VDS fish but significantly decreased *daglb* expression in liver. C: VDD fish had significantly increased *faah1* and *faah2a* expression in AT compared with VDS fish.

To support evidence of increased AEA synthesis in VDD fish compared with VDS fish, markers of EC signaling, synthesis, and metabolism were assessed using qPCR (Fig. 6). VDD fish exhibited attenuated receptor activation with reduced cannabinoid receptor 1 (*cnr1*), cannabinoid receptor 2 (*cnr2*), and transient receptor potential cation channel subfamily V member 1 (*trpv1*) expression in AT compared with VDS fish (Fig. 6A). VDD fish further demonstrated increased AEA synthesis with upregulated *N*-acyl phosphatidylethanolamine (PE) phospholipase D (*napepld*), alpha/beta hydrolase 4 (*abh4*), monoglyceride lipase (*mgll*), diacylglycerol lipase a/b (*dagla*/*b*), and glycerophosphodiester phosphodiesterase 1 (*gde1*) in AT compared with VDS fish (Fig. 6B). In liver, alterations in gene expression were less pronounced with the exception of a significant decrease in *daglb* in VDD fish compared with VDS fish (Fig. 6B). Finally, we found a striking increase in AEA metabolism demonstrated by increased fatty acid amide hydrolase 1 (*faah1*) and fatty acid amid hydrolase 2 (*faah2a*) expression in VDD AT compared with VDS fish (Fig. 6C).

## Discussion

In this study, we developed a novel lipidomics approach to establish whole-body lipid profiles in adult male zebrafish using two vitamin D dietary models. IR-MALDESI-MSI served as a powerful tool for capturing differences in lipid metabolite abundances and spatial distributions. After defining a specific ROI that incorporated key metabolic organs including AT, liver, intestines, spleen, and kidney, we utilized a series of data analysis tools including MSiReader, METLIN, and METASPACE, to discover and annotate ions that were significantly more abundant in the VDD fish. We chose to focus our investigation on putative identification of ions more abundant in the VDD fish compared with the VDS fish, given the robust fatty phenotype we previously published (35). Ultimately, we found that VDD zebrafish had a greater abundance of bioactive lipids compared with VDS zebrafish, suggestive of increased EC tone.

Previously our laboratory established a linkage between VDD and metabolic dyshomeostasis, where low levels of vitamin D results in an imbalance between energy utilization for somatic growth and energy storage in AT (35). We demonstrate that VDD disrupts the coordination of growth hormone (GH) and AT signaling via upregulation of suppressors of cytokine signaling (*socs*). This relationship suggested a direct link between VDD and disruption of GH signaling, where insufficient levels of vitamin D skewed the homeostatic regulation of somatic growth (anabolic GH activity) and adipose mobilization (catabolic GH activity). This current study served to further investigate a mechanistic linkage between VDD and metabolic disruption by using MS imaging to interrogate global changes within the zebrafish lipidome.

**Supplemental Figure 2:**
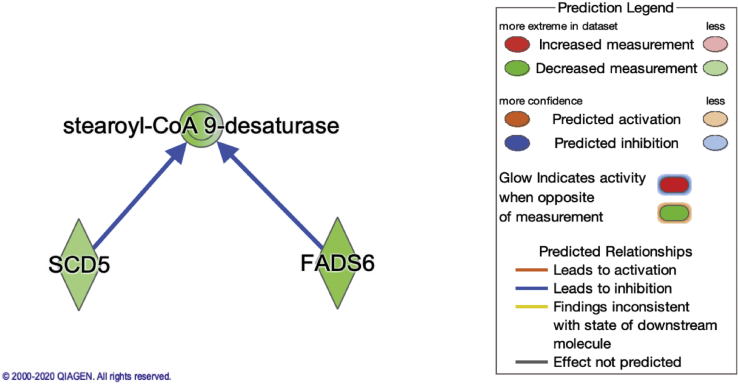


The EC system is a complex network of bioactive lipids ubiquitously expressed throughout the body (51). As previously mentioned, bioactive lipids are endogenous signaling lipids known to play critical roles in cellular function, including EC tone (22, 23). Included in this category are bile acids, eicosanoids, docosanoids, sphingolipids, and ECs. While not well investigated, preliminary studies suggest a potential linkage between VDD and metabolism of bioactive lipids including EC system modulation (24, 25). Like VDD, the EC system has been tightly linked to cardiovascular diseases, such as obesity, type 2 diabetes, liver disease, and metabolic syndrome (24, 33, 52, 53, 54, 55, 56). However, unlike VDD where BMI is inversely correlated with vitamin D levels, elevated levels of AEA and 2-arachidonoylglycerol (2-AG) are positively correlated with BMI and are consistently found elevated in obese individuals (24, 57, 58). Congruently, enhanced EC tone in AT is associated with fat accumulation (59). VDD fish presented greater levels of AEA, and thus enhanced EC tone, compared with VDS fish at 6 mpf. Elevated levels of AEA may be derived via elevated levels of fatty acyl precursors (arachidonic acid, eicosatetrienoic acid, and docosatrienoic acid) in the VDD fish (60). Increased arachidonic acid levels in particular have been directly linked to enhanced EC signaling and the development of obesity (52). Interestingly, in one particular study, elevated levels of arachidonic acid were detected in Wistar rats born to VDD mothers (25). In this study, authors suggested that attenuation of stearoyl-CoA 6-desaturase (Δ6-desaturase) and stearoyl-CoA 9-desaturase (Δ9-desaturase) mRNA levels could be contributing to the observed increase in arachidonic acid; however, the authors also suggest that mRNA abundance might not accurately reflect enzyme activity (25). Both desaturases play critical roles in catalyzing fatty acid desaturation, with Δ9-desaturase activity in particular suggested to protect against lipotoxicity (61, 62). We found that VDD fish not only exhibited attenuated Δ6-desaturase and Δ9-desaturase mRNA levels but also attenuated Δ5-desaturase mRNA levels as well, suggesting a potential role for vitamin D in desaturase modulation (supplemental Fig. S2).

Eicosatetrienoic acid, docosatrienoic acid, and arachidonic acid eventually feed into glycerolipid and glycerophospholipid synthesis, transforming into more complex lipids such as triglycerides and phosphatidylcholines (PCs), both of which are elevated in obese individuals (63, 64). PC and PE are important cellular membrane lipids, regulating critical lipogenic processes, such as lipoprotein secretion, lipid droplet formation, mitochondrial function, and cholesterol synthesis and secretion (65). Various cellular membrane lipids were detected in the interrogated ROI, including PCs and PEs; however, only two of these membrane lipids (PCs) were observed to be significantly elevated in the VDD zebrafish. An imbalance between PCs and PEs, where their molar ratio drops below 1.0, can lead to liver failure (65). Conversely, a molar ratio greater than 2.0 leads to nonalcoholic fatty liver disease or steatohepatitis and disrupted insulin signaling; however, in our VDD model, we do not see nonalcoholic fatty liver disease or steatohepatitis (65, 66). In addition to PCs, VDD fish exhibited elevated concentrations of numerous diacylglycerols and triacylglycerols compared with VDS fish. These findings can be supported by much of the literature describing EC signaling modulation of metabolic homeostasis, where enhanced EC signaling creates an environment favoring fat storage and weight gain, associated with diacylglycerol and triacylglycerol accumulation (28).

AEA and 2-AG activate two primary EC receptors CB1 and CB2; however, of the two receptors, CB1 plays the greatest role in regulation of metabolic homeostasis and has a greater presence in the liver and AT (67). CB2 is predominantly expressed in the spleen and activated by 2-AG and is suggested to play a greater role in immune function than energy metabolism (68). Activation of CB1 stimulates lipogenesis by promoting fatty acid synthesis, activating glucose transport into adipocytes, increasing expression of sterol regulatory element-binding protein-1c, and by decreasing mitochondrial activity through a reduction in proliferator activated receptor gamma coactivator 1α (*PPARGC1A*) (67, 69, 70, 71). Interestingly, we previously reported a significant increase in *srebf1* expression in the liver and AT, alongside a decrease in *ppargc1a* expression in the AT of VDD fish compared with VDS fish at 6 mpf (35). In zebrafish exposed to AEA, activation of CB1 is also linked to increased expression of insulin-like growth factors 1/2 (*igf1*/*igf2*); however, no association to adiposity is described (31). Previously, we reported significantly elevated levels of *igf1* and *igf2* in the liver, and elevated *igf1* levels in the AT, of VDD fish compared with VDS at 6 mpf (35). Similarly, *cyp2r1*^*−/−*^ fish, unable to activate vitamin D, demonstrated elevated levels of *igf1* in both the liver and AT (72). While inconsistent with truncated growth, elevated *igf1/igf2* levels have been linked to obesity and attenuation of lipolysis (73). Taken together, VDD may disrupt metabolic homeostasis through activation of EC signaling.

While AEA preferentially activates CB1, AEA can also activate peroxisome proliferator-activated receptor gamma (PPARγ) and transient receptor potential vanilloid 1 (TRPV1) directly (51, 53, 67, 69, 74, 75, 76). Activation of PPARγ by AEA leads to adipocyte differentiation and lipid accumulation (69). Our laboratory demonstrated significantly increased expression of *pparγ* in the liver and AT of VDD fish compared with VDS, suggesting a link between VDD and activation of *pparγ* via elevated levels of AEA (35). TRPV1 is in addition activated by AEA directly or indirectly via activation of CB1 by AEA (74). Activation of TRPV1 promotes AEA synthesis, mediates cellular uptake of AEA, and serves as an intracellular shuttle for AEA, collectively serving as a positive feedback loop (74, 76). During states of pancreatic inflammation, TRPV1 activation increases expression of calcitonin gene-related peptide, known to disrupt insulin release from pancreatic β-cells, promoting IR and obesity (74). Interestingly, preliminary data suggest TRPV1 as a target of vitamin D regulation in the pathogenesis of type 1 diabetes, with vitamin D acting as a TRPV1 inhibitor to reduce activation of naïve T cells (77). One theory is that VDD leads to activation of TRPV1, promoting AEA synthesis, and enhanced EC signaling. We found attenuated levels of *cnr1*, *cnr2*, and *trpv1* in VDD AT compared with VDS AT. This may suggest a compensatory downregulation or desensitization of these receptors in response to excessive levels of AEA found in the VDD fish. Furthermore, we found increased expression of genes responsible for AEA synthesis (*napepld*, *abh4*, *dagla*/*b*, *mgll*, and *gde1*) and AEA metabolism (*faah1* and *faah2a*) in VDD AT, supporting increased levels of AEA in VDD fish compared with VDS fish. However, we did not see the same increase in AEA synthesis in the liver based on gene expression changes, suggesting that our observed increase in AEA is more likely a result of activity in the AT.

Select bile acids/alcohols and vitamin D derivatives were also upregulated in VDD zebrafish. Elevated levels of bile acids and vitamin D derivatives could indicate enhanced cholesterol turnover in the VDD fish as a compensatory mechanism for low vitamin D levels. Bile acids are end-stage cholesterol metabolites that aid in digestion (78, 79). In particular, 5β-cyprinolsulfate was detected in our dataset. 5β-cyprinolsulfate is the major bile salt found in zebrafish; however, the exact reason behind a greater abundance of 5β-cyprinolsulfate in the VDD fish is unclear without further investigation (80). Several vitamin D derivatives were also elevated in our VDD dataset. Previously, vitamin D levels of calcidiol (25(OH)D3) and calcitriol (1,25(OH)2D3) were measured in our VDD fish and found to be significantly decreased (35). The derivatives found in this dataset putatively indicate an alternative or nontraditional synthesis pathway being stimulated in the VDD fish as a compensatory mechanism for the VDD. Further work is needed to determine the biological impact of the varying derivatives.

In our model, we demonstrate a relationship between VDD and enhanced EC signaling, where low levels of vitamin D are associated with increased production of bioactive lipids. This relationship suggests that VDD may disrupt metabolic homeostasis through promoting synthesis of bioactive lipids known to favor fat storage. These data demonstrate a putative linkage between VDD and EC signaling, a novel finding in the field of metabolic health. Future work aimed to investigate the inhibitory role of vitamin D on TRPV1 activation by AEA would provide greater insight on the mechanistic underpinnings of this relationship. Finally, we chose to focus this study on ions more abundant in the VDD fish given their robust fatty phenotype, but future investigations aim to identify ions more abundant in the VDS zebrafish that could potentially be protective against VDD-induced adiposity.

## Data availability

The MS imaging data are public at https://metaspace2020.eu/project/Knuth_et_al_2021. The RNA-Seq data referenced in supplemental data (supplemental Fig. S2) were previously made public through Mendeley Data: Knuth, Megan; Kullman, Seth (2020), “Vitamin D deficiency serves as a precursor to stunted growth and central adiposity_Knuth *et al.*,” Mendeley Data, V1, https://doi.org/10.17632/6k7x7twz48.1.

## Supplemental data

This article contains supplemental data.

## Conflict of interest

The authors declare that they have no conflicts of interest with the contents of this article.

## Figures and Tables

**Supplemental Table 1:**
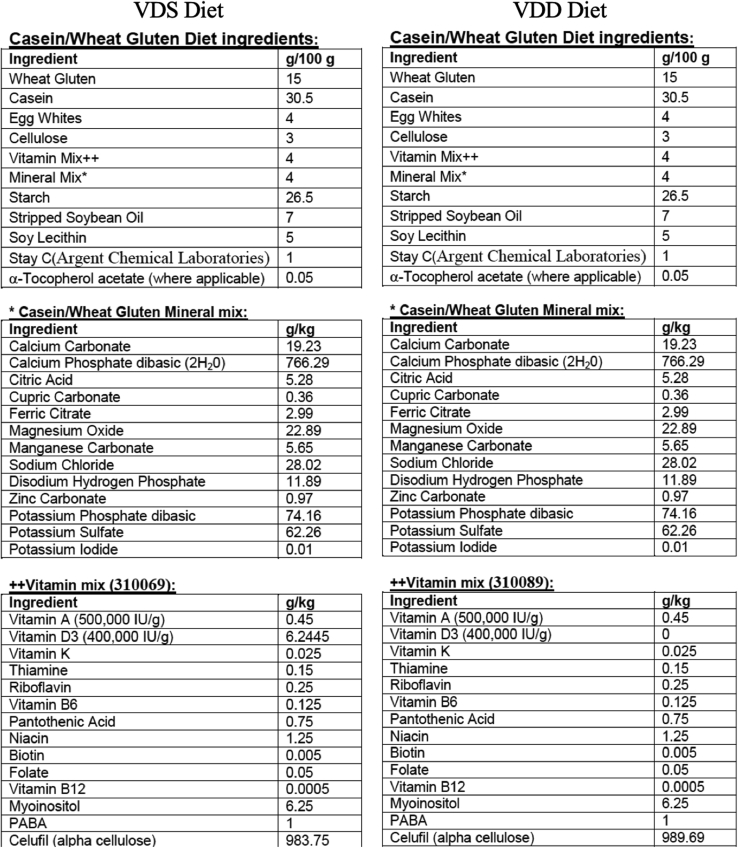


## References

[1] Ross A.C., Taylor C.L., Yaktine A.L., Valle H.B.D., Institute of Medicine (US) Committee to Review Dietary Reference Intakes for Vitamin D and Calcium (2011).

[2] Bouillon R., Suda T. (2014). Vitamin D: calcium and bone homeostasis during evolution. Bonekey Rep..

[3] Veldurthy V., Wei R., Oz L., Dhawan P., Jeon Y.H., Christakos S. (2016). Vitamin D, calcium homeostasis and aging. Bone Res..

[4] Abbas M.A. (2017). Physiological functions of vitamin D in adipose tissue. J. Steroid Biochem. Mol. Biol..

[5] Eyles D.W., Burne T.H., McGrath J.J. (2013). Vitamin D, effects on brain development, adult brain function and the links between low levels of vitamin D and neuropsychiatric disease. Front. Neuroendocrinol..

[6] Mora J.R., Iwata M., von Andrian U.H. (2008). Vitamin effects on the immune system: vitamins A and D take centre stage. Nat. Rev. Immunol..

[7] Asano L., Watanabe M., Ryoden Y., Usuda K., Yamaguchi T., Khambu B., Takashima M., Sato S.I., Sakai J., Nagasawa K., Uesugi M. (2017). Vitamin D metabolite, 25-hydroxyvitamin D, regulates lipid metabolism by inducing degradation of SREBP/SCAP. Cell Chem. Biol..

[8] Aranow C. (2011). Vitamin D and the immune system. J. Investig. Med..

[9] Santos G.C., Zeidler J.D., Pérez-Valencia J.A., Sant'Anna-Silva A.C.B., Da Poian A.T., El-Bacha T., Almeida F.C.L. (2017). Metabolomic analysis reveals vitamin D-induced decrease in polyol pathway and subtle modulation of glycolysis in HEK293T cells. Sci. Rep..

[10] Ideraabdullah F.Y., Belenchia A.M., Rosenfeld C.S., Kullman S.W., Knuth M., Mahapatra D., Bereman M., Levin E.D., Peterson C.A. (2019). Maternal vitamin D deficiency and developmental origins of health and disease (DOHaD). J. Endocrinol..

[11] Krishnaveni G.V., Veena S.R., Winder N.R., Hill J.C., Noonan K., Boucher B.J., Karat S.C., Fall C.H. (2011). Maternal vitamin D status during pregnancy and body composition and cardiovascular risk markers in Indian children: the Mysore Parthenon Study. Am. J. Clin. Nutr..

[12] Crozier S.R., Harvey N.C., Inskip H.M., Godfrey K.M., Cooper C., Robinson S.M., SWS Study Group (2012). Maternal vitamin D status in pregnancy is associated with adiposity in the offspring: findings from the Southampton Women’s Survey. Am. J. Clin. Nutr..

[13] Hawes J.E., Tesic D., Whitehouse A.J., Zosky G.R., Smith J.T., Wyrwoll C.S. (2015). Maternal vitamin D deficiency alters fetal brain development in the BALB/c mouse. Behav. Brain Res..

[14] Maia-Ceciliano T.C., Barreto-Vianna A.R., Barbosa-da-Silva S., Aguila M.B., Faria T.S., Mandarim-de-Lacerda C.A. (2016). Maternal vitamin D-restricted diet has consequences in the formation of pancreatic islet/insulin-signaling in the adult offspring of mice. Endocrine.

[15] Pramono A., Jocken J.W.E., Essers Y.P.G., Goossens G.H., Blaak E.E. (2019). Vitamin D and tissue-specific insulin sensitivity in humans with overweight/obesity. J. Clin. Endocrinol. Metab..

[16] Aatsinki S.M., Elkhwanky M.S., Kummu O., Karpale M., Buler M., Viitala P., Rinne V., Mutikainen M., Tavi P., Franko A., Wiesner R.J., Chambers K.T., Finck B.N., Hakkola J. (2019). Fasting-induced transcription factors repress vitamin D bioactivation, a mechanism for vitamin D deficiency in diabetes. Diabetes.

[17] Rocha L.M., Baldan D.C.d.S., Souza A.L., Chaim E.A., Pavin E.J., Alegre S.M. (2017). Body composition and metabolic profile in adults with vitamin D deficiency. Rev. Nutr..

[18] Lerchbaum E., Trummer C., Theiler-Schwetz V., Kollmann M., Wölfler M., Pilz S., Obermayer-Pietsch B. (2019). Effects of vitamin D supplementation on body composition and metabolic risk factors in men: a randomized controlled trial. Nutrients.

[19] Litonjua A.A., Carey V.J., Laranjo N., Harshfield B.J., McElrath T.F., O'Connor G.T., Sandel M., Iverson R.E., Lee-Paritz A., Strunk R.C., Bacharier L.B., Macones G.A., Zeiger R.S., Schatz M., Hollis B.W. (2016). Effect of prenatal supplementation with vitamin D on asthma or recurrent wheezing in offspring by age 3 years: the VDAART randomized clinical trial. JAMA.

[20] Blighe K., Chawes B.L., Kelly R.S., Mirzakhani H., McGeachie M., Litonjua A.A., Weiss S.T., Lasky-Su J.A. (2017). Vitamin D prenatal programming of childhood metabolomics profiles at age 3 y. Am. J. Clin. Nutr..

[21] Boden G. (2008). Obesity and free fatty acids (FFA). Endocrinol. Metab. Clin. North Am..

[22] Wasserman A.H., Venkatesan M., Aguirre A. (2020). Bioactive lipid signaling in cardiovascular disease, development, and regeneration. Cells.

[23] Chiurchiù V., Leuti A., Maccarrone M. (2018). Bioactive lipids and chronic inflammation: managing the fire within. Front. Immunol..

[24] Di Marzo V., Silvestri C. (2019). Lifestyle and metabolic syndrome: contribution of the endocannabinoidome. Nutrients.

[25] Nandi A., Wadhwani N., Joshi S.R. (2019). Vitamin D deficiency influences fatty acid metabolism. Prostaglandins Leukot. Essent. Fatty Acids.

[26] Guida F., Boccella S., Belardo C., Iannotta M., Piscitelli F., De Filippis F., Paino S., Ricciardi F., Siniscalco D., Marabese I., Luongo L., Ercolini D., Di Marzo V., Maione S. (2020). Altered gut microbiota and endocannabinoid system tone in vitamin D deficiency-mediated chronic pain. Brain Behav. Immun..

[27] Sophocleous A., Robertson R., Ferreira N.B., McKenzie J., Fraser W.D., Ralston S.H. (2017). Heavy cannabis use is associated with low bone mineral density and an increased risk of fractures. Am. J. Med..

[28] van Eenige R., van der Stelt M., Rensen P.C.N., Kooijman S. (2018). Regulation of adipose tissue metabolism by the endocannabinoid system. Trends Endocrinol. Metab..

[29] Aguirre C.A., Castillo V.A., Llanos M.N. (2015). The endocannabinoid anandamide during lactation increases body fat content and CB1 receptor levels in mice adipose tissue. Nutr. Diabetes.

[30] Liu L.Y., Alexa K., Cortes M., Schatzman-Bone S., Kim A.J., Mukhopadhyay B., Cinar R., Kunos G., North T.E., Goessling W. (2016). Cannabinoid receptor signaling regulates liver development and metabolism. Development.

[31] Migliarini B., Carnevali O. (2008). Anandamide modulates growth and lipid metabolism in the zebrafish Danio rerio. Mol. Cell. Endocrinol..

[32] Pai W.Y., Hsu C.C., Lai C.Y., Chang T.Z., Tsai Y.L., Her G.M. (2013). Cannabinoid receptor 1 promotes hepatic lipid accumulation and lipotoxicity through the induction of SREBP-1c expression in zebrafish. Transgenic Res..

[33] Engeli S., Böhnke J., Feldpausch M., Gorzelniak K., Janke J., Bátkai S., Pacher P., Harvey-White J., Luft F.C., Sharma A.M., Jordan J. (2005). Activation of the peripheral endocannabinoid system in human obesity. Diabetes.

[34] Xuan L., Ma J., Yu M., Yang Z., Huang Y., Guo C., Lu Y., Yan L., Sh S. (2019). Insulin-like growth factor 2 promotes adipocyte proliferation, differentiation and lipid deposition in obese type 2 diabetes. J. Transl. Sci..

[35] Knuth M.M., Mahapatra D., Jima D., Wan D., Hammock B.D., Law M., Kullman S.W. (2020). Vitamin D deficiency serves as a precursor to stunted growth and central adiposity in zebrafish. Sci. Rep..

[36] Robichaud G., Barry J.A., Garrard K.P., Muddiman D.C. (2013). Infrared matrix-assisted laser desorption electrospray ionization (IR-MALDESI) imaging source coupled to a FT-ICR mass spectrometer. J. Am. Soc. Mass Spectrom..

[37] Stutts W.L., Knuth M.M., Ekelöf M., Mahapatra D., Kullman S.W., Muddiman D.C. (2020). Methods for cryosectioning and mass spectrometry imaging of whole-body zebrafish. J. Am. Soc. Mass Spectrom..

[38] Bokhart M.T., Nazari M., Garrard K.P., Muddiman D.C. (2018). MSiReader v1.0: evolving open-source mass spectrometry imaging software for targeted and untargeted analyses. J. Am. Soc. Mass Spectrom..

[39] Robichaud G., Garrard K.P., Barry J.A., Muddiman D.C. (2013). MSiReader: an open-source interface to view and analyze high resolving power MS imaging files on Matlab platform. J. Am. Soc. Mass Spectrom..

[40] Palmer A., Phapale P., Chernyavsky I., Lavigne R., Fay D., Tarasov A., Kovalev V., Fuchser J., Nikolenko S., Pineau C., Becker M., Alexandrov T. (2017). FDR-controlled metabolite annotation for high-resolution imaging mass spectrometry. Nat. Methods.

[41] Smith C.A., O'Maille G., Want E.J., Qin C., Trauger S.A., Brandon T.R., Custodio D.E., Abagyan R., Siuzdak G. (2005). METLIN: a metabolite mass spectral database. Ther. Drug Monit..

[42] Sud M., Fahy E., Cotter D., Brown A., Dennis E.A., Glass C.K., Merrill A.H., Murphy R.C., Raetz C.R., Russell D.W., Subramaniam S. (2007). LMSD: LIPID MAPS structure database. Nucleic Acids Res..

[43] Ruttkies C., Schymanski E.L., Wolf S., Hollender J., Neumann S. (2016). MetFrag relaunched: incorporating strategies beyond in silico fragmentation. J. Cheminform..

[44] Livak K.J., Schmittgen T.D. (2001). Analysis of relative gene expression data using real-time quantitative PCR and the 2−ΔΔCT method. Methods.

[45] Watson A.T., Planchart A., Mattingly C.J., Winkler C., Reif D.M., Kullman S.W. (2017). From the cover: embryonic exposure to TCDD impacts osteogenesis of the axial skeleton in Japanese medaka, Oryzias latipes. Toxicol. Sci..

[46] Ovchinnikova K., Kovalev V., Stuart L., Alexandrov T. (2019). Recognizing off-sample mass spectrometry images with machine and deep learning. bioRxiv.

[47] Zhou W., Hong Y., Huang C., Shen C., Chu Y. (2019). Laser ablation electrospray ionization time-of-flight mass spectrometry for direct analysis of biological tissue. J. Anal. Methods Chem..

[48] Guenther S., Schäfer K.C., Balog J., Dénes J., Majoros T., Albrecht K., Tóth M., Spengler B., Takáts Z. (2011). Electrospray post-ionization mass spectrometry of electrosurgical aerosols. J. Am. Soc. Mass Spectrom..

[49] Bagley M.C., Ekelöf M., Muddiman D.C. (2020). Determination of optimal electrospray parameters for lipidomics in infrared-matrix-assisted laser desorption electrospray ionization mass spectrometry imaging. J. Am. Soc. Mass Spectrom..

[50] Bai H., Linder K.E., Muddiman D.C. (2021). Three-dimensional (3D) imaging of lipids in skin tissues with infrared matrix-assisted laser desorption electrospray ionization (MALDESI) mass spectrometry. Anal. Bioanal. Chem..

[51] Maccarrone M. (2017). Metabolism of the endocannabinoid anandamide: open questions after 25 years. Front. Mol. Neurosci..

[52] Alvheim A.R., Malde M.K., Osei-Hyiaman D., Lin Y.H., Pawlosky R.J., Madsen L., Kristiansen K., Frøyland L., Hibbeln J.R. (2012). Dietary linoleic acid elevates endogenous 2-AG and anandamide and induces obesity. Obesity (Silver Spring).

[53] Di Marzo V. (2008). The endocannabinoid system in obesity and type 2 diabetes. Diabetologia.

[54] Rossi F., Punzo F., Umano G.R., Argenziano M., Miraglia Del Giudice E. (2018). Role of cannabinoids in obesity. Int. J. Mol. Sci..

[55] Basu P.P., Aloysius M.M., Shah N.J., Brown R.S. (2014). Review article: the endocannabinoid system in liver disease, a potential therapeutic target. Aliment. Pharmacol. Ther..

[56] Bazwinsky-Wutschke I., Zipprich A., Dehghani F. (2019). Endocannabinoid system in hepatic glucose metabolism, fatty liver disease, and cirrhosis. Int. J. Mol. Sci..

[57] Matias I., Gatta-Cherifi B., Tabarin A., Clark S., Leste-Lasserre T., Marsicano G., Piazza P.V., Cota D. (2012). Endocannabinoids measurement in human saliva as potential biomarker of obesity. PLoS One.

[58] Bluher M., Engeli S., Klöting N., Berndt J., Fasshauer M., Bátkai S., Pacher P., Schön M.R., Jordan J., Stumvoll M. (2006). Dysregulation of the peripheral and adipose tissue endocannabinoid system in human abdominal obesity. Diabetes.

[59] Izzo A.A., Piscitelli F., Capasso R., Aviello G., Romano B., Borrelli F., Petrosino S., Di Marzo V. (2009). Peripheral endocannabinoid dysregulation in obesity: relation to intestinal motility and energy processing induced by food deprivation and re-feeding. Br. J. Pharmacol..

[60] Naughton S.S., Mathai M.L., Hryciw D.H., McAinch A.J. (2013). Fatty acid modulation of the endocannabinoid system and the effect on food intake and metabolism. Int. J. Endocrinol..

[61] Dalla Valle A., Vertongen P., Spruyt D., Lechanteur J., Suain V., Gaspard N., Brion J.P., Gangji V., Rasschaert J. (2019). Induction of stearoyl-CoA 9-desaturase 1 protects human mesenchymal stromal cells against palmitic acid-induced lipotoxicity and inflammation. Front. Endocrinol. (Lausanne).

[62] Bond L.M., Miyazaki M., O’Neill L.M., Ding F., Ntambi J.M. (2016). Biochemistry of Lipids, Lipoproteins and Membranes.

[63] Whelan J., Fritsche K. (2013). Linoleic acid. Adv. Nutr..

[64] Ahmadian M., Duncan R.E., Jaworski K., Sarkadi-Nagy E., Sul H.S. (2007). Triacylglycerol metabolism in adipose tissue. Future Lipidol..

[65] van der Veen J.N., Kennelly J.P., Wan S., Vance J.E., Vance D.E., Jacobs R.L. (2017). The critical role of phosphatidylcholine and phosphatidylethanolamine metabolism in health and disease. Biochim. Biophys. Acta Biomembr..

[66] Li Z., Agellon L.B., Allen T.M., Umeda M., Jewell L., Mason A., Vance D.E. (2006). The ratio of phosphatidylcholine to phosphatidylethanolamine influences membrane integrity and steatohepatitis. Cell Metab..

[67] Vettor R., Pagano C. (2009). The role of the endocannabinoid system in lipogenesis and fatty acid metabolism. Best Pract. Res. Clin. Endocrinol. Metab..

[68] Oltrabella F., Melgoza A., Nguyen B., Guo S. (2017). Role of the endocannabinoid system in vertebrates: emphasis on the zebrafish model. Dev. Growth Differ..

[69] Matias I., Belluomo I., Cota D. (2016). The fat side of the endocannabinoid system: role of endocannabinoids in the adipocyte. Cannabis Cannabinoid Res..

[70] Ruiz de Azua I., Mancini G., Srivastava R.K., Rey A.A., Cardinal P., Tedesco L., Zingaretti C.M., Sassmann A., Quarta C., Schwitter C., Conrad A., Wettschureck N., Vemuri V.K., Makriyannis A., Hartwig J. (2017). Adipocyte cannabinoid receptor CB1 regulates energy homeostasis and alternatively activated macrophages. J. Clin. Invest..

[71] Tedesco L., Valerio A., Dossena M., Cardile A., Ragni M., Pagano C., Pagotto U., Carruba M.O., Vettor R., Nisoli E. (2010). Cannabinoid receptor stimulation impairs mitochondrial biogenesis in mouse white adipose tissue, muscle, and liver: the role of eNOS, p38 MAPK, and AMPK pathways. Diabetes.

[72] Peng X., Shang G., Wang W., Chen X., Lou Q., Zhai G., Li D., Du Z., Ye Y., Jin X., He J., Zhang Y., Yin Z. (2017). Fatty acid oxidation in zebrafish adipose tissue is promoted by 1α,25(OH)2D3. Cell Rep..

[73] AsghariHanjani N., Vafa M. (2019). The role of IGF-1 in obesity, cardiovascular disease, and cancer. Med. J. Islam Repub. Iran.

[74] Christie S., Wittert G.A., Li H., Page A.J. (2018). Involvement of TRPV1 channels in energy homeostasis. Front. Endocrinol. (Lausanne).

[75] Tóth A., Blumberg P.M., Boczán J. (2009).

[76] Hofmann N.A., Barth S., Waldeck-Weiermair M., Klec C., Strunk D., Malli R., Graier W.F. (2014). TRPV1 mediates cellular uptake of anandamide and thus promotes endothelial cell proliferation and network-formation. Biol. Open.

[77] Long W., Fatehi M., Barr A., Kelly R., Held M., Baldwin Y.Y., Light P. (2017). Vitamin D directly inhibits TRPV1 channels and reduces the activation of naïve T-cells. Can. J. Diabetes.

[78] Ma H., Patti M.E. (2014). Bile acids, obesity, and the metabolic syndrome. Best Pract. Res. Clin. Gastroenterol..

[79] Hagey L.R., Møller P.R., Hofmann A.F., Krasowski M.D. (2010). Diversity of bile salts in fish and amphibians: evolution of a complex biochemical pathway. Physiol. Biochem. Zool..

[80] Kurogi K., Yoshihama M., Horton A., Schiefer I.T., Krasowski M.D., Hagey L.R., Williams F.E., Sakakibara Y., Kenmochi N., Suiko M., Liu M.C. (2017). Identification and characterization of 5α-cyprinol-sulfating cytosolic sulfotransferases (Sults) in the zebrafish (Danio rerio). J. Steroid Biochem. Mol. Biol..

