# Supplementary material for: Vitamin D deficiency promotes accumulation of bioactive lipids and increased endocannabinoid tone in zebrafish
Source: J Lipid Res. 2021 Oct 18;62:100142. doi: 10.1016/j.jlr.2021.100142 (PMC8604674; doi:10.1016/j.jlr.2021.100142)
Supplement: Supplemental data [file mmc1.docx]

**Vitamin D deficiency promotes accumulation of bioactive lipids and increased endocannabinoid tone in zebrafish.**

Megan M. Knuth^1,2*^, Whitney L. Stutts^3^, Morgan M. Ritter^4^, Kenneth P. Garrard^3,5,6^, Seth W. Kullman^4,7^

^1^Lineberger Comprehensive Cancer Center, University of North Carolina School of Medicine at Chapel Hill, Chapel Hill, NC 27514, USA.

^2^Department of Genetics, University of North Carolina School of Medicine at Chapel Hill, Chapel Hill, NC 27514, USA.

^3^Molecular Education, Technology and Research Innovation Center (METRIC), North Carolina State University, Raleigh, NC 27606, USA.

^4^Toxicology Program, Department of Biological Sciences, North Carolina State University, Raleigh, NC 27606, USA.

^5^FTMS Laboratory for Human Health Research and Department of Chemistry, North Carolina State University, Raleigh, NC 27607, USA.

^6^Precision Engineering Consortium, Department of Mechanical and Aerospace Engineering, North Carolina State University, Raleigh, NC 27695, USA.

^7^Center for Human Health and the Environment, North Carolina State University, Raleigh, NC 27606, USA.

*Corresponding author: Megan Knuth, mmknuth@email.unc.edu, mailing address - Campus Box 7264, Chapel Hill, NC 27514, telephone 919.602.2097

Short Title: **Vitamin D deficiency promotes accumulation of bioactive lipids.**

Funding: This work was supported by the National Institute of Environmental Health Sciences: *T32ES07046* *Molecular Pathways to Pathogenesis in Toxicology*, *P30ES025128*, and the Environmental Protection Agency STAR *RD-83342002*.

Keywords: mass spectrometry, metabolomics, lipidomics, lipids, nutrition**Supplemental Data**

**Materials and Methods**

*RNA Seq*

As previously described, illumina RNA library construction and RNA sequencing was completed at the NC State Genomic Sciences Laboratory (Raleigh, NC).^35^ Prior to library construction, total RNA was extracted from vitamin D deficient, sufficient, and control male zebrafish livers (n=4/diet), and analyzed for purity, concentration, and integrity. Purification of mRNA was performed using the oligo-dT beads provided in the NEBNExt Poly(A) mRNA Magnetic Isolation Module (New England Biolabs, Ipswich, MA). Complementary DNA (cDNA) libraries for Illumina sequencing were constructed using the NEBNext Ultra Directional RNA Library Prep Kit (NEB) and NEBNext Mulitplex Oligos for Illumina (NEB) using the manufacturer-specified protocol. Briefly, mRNA was chemically fragmented and primed with random oligos for first strand cDNA synthesis. Second strand cDNA synthesis was then carried out with dUTPs to preserve strand orientation information. The double-stranded cDNA was then purified, end repaired and “a-tailed” for adaptor ligation. Following ligation, the samples were selected from a final library size of 400–550 bp using sequential AMPure XP bead isolation (Beckman Coulter, USA). Library enrichment was performed and specific indexes for each sample were added during the protocol-specified PCR amplification. The amplified library fragments were purified and checked for quality and final concentration. The final quantified libraries were pooled in equimolar amounts for clustering and sequencing on an Illumina HiSeq 2500 DNA Sequencer, utilizing a 125 bp single end sequencing reagent kit (Illumina, USA). Data analysis was performed in consultation with Bioinformatics Core at NCSU Center for Human Health and the Environment. Ingenuity Pathway Analysis (IPA) version 01-10(01-10) provided predictive models of pathway modulation based on transcript abundance (VDD vs. VDS). Using the ‘Genes and Chemicals’ tool, stearoyl-CoA desaturases 5, 6, and 9 were loaded separately into the ‘Canonical Pathways’ window. Within the ‘Canonical Pathways’ window, overlay and molecule activity predictor (MAP) functions were applied and the fold change (increased, decreased) of each desaturase was represented as VDD/VDS.

**Supplemental Table Titles and Legends**

**S Table 1**. Nutritional breakdown of VDS and VDD diets.

**S Table 2**. qPCR primer list

**S Table 2**. Gene expression was normalized to *efla* as the housekeeping gene.

**Supplemental Figure Legends**

**S Figure 1**. Data analysis workflow in MSiReader for pipeline A. (A) MSiReader window demonstrating the selection of ROIs within each treatment group. (B) MSi Peakfinder settings used to export all peaks 2-fold or greater in the VDD fish ROIs than the VDS fish ROIs. (C) MSi Export window used to export abundance data for selected pixels in (A).

**S Figure 2**. RNA-Seq IPA (version 01-10(01-10)) analysis of liver mRNA abundance. Stearoyl-CoA desaturases 5, 6, and 9 are all decreased in VDD fish compared to VDS fish 6mpf. Fold change (increased, decreased) determined as VDD/VDS.

https://analysis.ingenuity.com/pa/launch.jsp
