# Supplementary material for: Vitamin D deficiency promotes accumulation of bioactive lipids and increased endocannabinoid tone in zebrafish
Source: J Lipid Res. 2021 Oct 18;62:100142. doi: 10.1016/j.jlr.2021.100142 (PMC8604674; doi:10.1016/j.jlr.2021.100142)
Supplement: Supplemental Table 2 [file mmc2.docx]

| **Gene** | **Primer Sequence (5’- 3’)** | **Product Size (bp**) |
| --- | --- | --- |
| *ef1a* | TACAAATGCGGTGGAATCGAC (forward)  GTCAGCCTGAGAAGTACCAGT (reverse) | 246 |
| *abh4* | CAAATCAAACCAGAATATGGACCCTT (forward)  GCATCCAGATTACGAATCCAGAGG (reverse) | 139 |
| *cnr1* | TCTGTGGGAAGCCTGTTT (forward)  ACCGAGTTGAGCCGTTTG (reverse) | 182 |
| *cnr2* | GCAGAGCGTGAAAGGACAG (forward)  GATCGCCAGGATTAGAAGGA (reverse) | 85 |
| *dagla* | CAGCCATCTTGGATCAGGCAA (forward)  ATAAACGGGCCAGCCATATGC (reverse) | 170 |
| *daglb* | CTTCATGGACACAGATCTGGT (forward)  TATGGGAGAGGACGGACTG (reverse) | 130 |
| *faah1* | TACTAAACGCGCGGAGAAGA (forward)  AGTGAGCTCCGACAAAGACA (reverse) | 128 |
| *faah2a* | CAGGACCGATGTGCCGTTAT (forward)  CCACTTCAGTAAAGAGAGACAGTTTC (reverse) | 96 |
| *gde1* | CTGCTATAAGAGCGGCGAGT (forward)  CCACGGTTTCATCGTGCATC (reverse) | 102 |
| *mgll* | GGGATCCCAAACAGGTGGAG (forward)  AAAGGGCCACCTGATGTCTG (reverse) | 137 |
| *napepld* | TTTGACTTGGCTGCAATCCC (forward)  TCGTGCTGGAGGTTCAAGAT (reverse) | 180 |
| *trpv1* | TCCAACCCTCAAAGTCGTATG (forward)  TCAATCCAAATCGTCCCCTG (reverse) | 73 |
